# Editorial: Soft ticks as parasites and vectors

**DOI:** 10.3389/fvets.2022.1037492

**Published:** 2022-10-31

**Authors:** Ben J. Mans, José M. Venzal, Sebastián Muñoz-Leal

**Affiliations:** ^1^Epidemiology, Parasites and Vectors, Agricultural Research Council, Pretoria, South Africa; ^2^Department of Veterinary Tropical Diseases, University of Pretoria, Pretoria, South Africa; ^3^Department of Life and Consumer Sciences, University of South Africa, Pretoria, South Africa; ^4^Centro Universitario Regional (CENUR) Litoral Norte, Universidad de la República, Salto, Uruguay; ^5^Department of Animal Science, University of Concepcion, Chillán, Chile

**Keywords:** argasids, ecology, bloodmeal, salivary glands, distribution

Soft ticks are interesting ectoparasites due to their ecological habits and unique blood-feeding biology. The wide-ranging nature of research into these parasites is reflected in the diverse set of papers captured in this special issue. Fundamental questions are addressed in the 9 articles included in this Research Topic that focuses on ecology, tick-host interaction, host associations, geographic distribution, and microbial endosymbionts.

## Geographic dispersal of soft ticks

Adult and nymphal soft ticks feed quickly and are therefore not extensively associated with their hosts. In addition, they are generally nest- or burrow-dwelling parasites. Therefore, how they disperse to new hosts and burrows is an interesting question to tackle. The study by Rataud et al. investigated dispersal between yellow-legged gull (*Larus michahellis*) nests for the soft tick *Ornithodoros maritimus* (also known as *Alectorobius maritimus*) using a capture-mark-recapture strategy within a natural bird population during the breeding season. Overall dispersal rates were low, confirming the strong endophilic nature of this tick species. This contrasted with previous results on the random distribution of infectious organisms in this species that suggested extensive between-nest movement (1). The study considered the possibility that longer temporal scales of dispersal may be more relevant to argasid dispersal than short-term monitoring allows. The study certainly highlights great mysteries in soft tick biology that exist regarding geographic and temporal dispersal, and how life stage, seasonality, nest- or burrow-occupancy, and tick-feeding status may impact this.

## Host identification through bloodmeal analysis

The ability of soft ticks to survive for extensive periods of time is linked with their slow metabolism and capability to store their bloodmeal for prolonged periods in an intact and undigested form. The fact that soft ticks can feed multiple times and that the previous bloodmeals survive feeding events in their guts offers the opportunity to utilize the bloodmeal to identify past hosts. This biological particularity would greatly expand the ability to associate soft ticks with their natural hosts, since the ticks are rarely collected on the host or dwell in burrows that harbor multiple vertebrate species, making host identification ambiguous. Two studies in this collection investigated various methods to detect hosts by bloodmeal analysis of the soft tick *Ornithodoros (Pavlovskyella) turicata*. On the one hand, the study by Busselman et al. validated the use of PCR and Sanger sequencing in ticks artificially fed on chicken and pig blood and could detect host DNA up to 1,105 days after feeding. Based on these parameters, they screened 19 field-collected ticks from a cave in Austin, Texas, and showed evidence of the presence of raccoon, black vulture, Texas black rattlesnake, and human blood in bloodmeals. On the other hand, the study by Kim et al. investigated species-specific qPCR analysis using ticks fed on various host species such as chicken, goat, and pig. According to their results, the specific origin of bloodmeals could be detected beyond 330 days post-feeding, through multiple molting events and multiple species in one bloodmeal. The authors also investigated the use of stable isotope analyses for the identification of bloodmeal origin in ticks, but their results suggest that this approach is less sensitive than qPCR. These studies highlighted the potential to use bloodmeal identification analyses as means to detect prior hosts and exploit the ability of soft ticks to retain intact host blood across molts and feeding events. While the strategies used, such as host-specific qPCR and direct Sanger sequencing may limit the ability to detect multiple hosts, future prospects such as cloning and sequencing of amplicons or implementing next-generation amplicon sequencing approaches will expand the opportunity to uncover the array of hosts parasitized by soft ticks in their natural habitats.

## Domestic fowl as hosts of soft ticks

Most argasids parasitize wild animals and their impact on domestic animal or human health may be considered incidental, as found for the human that visited the cave frequented by *O. (P.) turicata* above (Busselman et al.). However, when infestations of argasids are experienced in human homes or domestic animal dwellings, the impact can be quite severe, especially when infected with pathogenic agents. For instance, *Argas persicus* parasitize poultry and has a worldwide distribution with a preference for tropical regions, and is generally the expected tick species when poultry dwellings are investigated. In the current collection, two studies geographically distant from each other investigated the infestation of poultry by argasids. Zahid et al. studied the infestation of domestic fowl in Khyber Pakhtunkhwa, Pakistan. From a large number of ticks collected from fowl dwellings (*n* = 7,219), the only tick found was *A. persicus*, as confirmed by morphological and molecular identification. The second study by López et al. from the Caribbean region of Colombia surprisingly found the exclusive infestation of fowl dwellings by *Ornithodoros puertoricensis* (also known as *Alectorobius puertoricensis*) as confirmed by morphological and molecular identification. This was the first association of this tick species with domestic fowl. Both studies highlight the importance of confirming which tick species parasitize domestic fowl in a given region since this will also impact on risk assessment of zoonosis and the possibility that high infestations can spill over to nearby human dwellings.

## Birds as hosts of soft ticks

Palomar et al. collected soft ticks in wild birds from Spain. In this study, the authors were unable to identify the ticks to the species level using current available morphological keys. Species relationships were further investigated using mitochondrial 12S and 16S ribosomal RNA and cytochrome oxidase 1 genes. Phylogenetic analysis of the 16S rRNA indicated species identity with *A. persicus* and *Argas reflexus*, with at least two genotypes grouping with a clade formed by *Argas japonicus, Argas lagenoplastis, Argas polonicus*, and *Argas vulgaris* and a genotype that grouped with *Argas africolumbae*. The study pointed out the important need for the development of accurate keys for the genus *Argas*, given that this genus comprises 44 species with many potential new *Argas* species, as found in the Palearctic region. The study also detected *Rickettsia, Coxiella, Francisella*, and *Rickettsiella* species as well as a novel *Babesia* genotype, closely related to avian *Babesia* species. This highlights the potential role of *Argas* species in transmitting zoonotic bacterial and piroplasmid agents.

## Bat-associated soft ticks

Sándor et al. investigated the associations between soft ticks and hosts, using mammals as study models. Five main tick species were identified, namely, *Carios vespertilionis, Chiropterargas boueti, Chiropterargas confusus, Reticulinasus salahi*, and *Secretargas transgariepinus*. Except for *C. vespertilionis* which showed the widest distribution, the distribution maps for these ticks are data sparse and indicate a need for more studies on bats and their associated argasid species. The maps presented will serve as important reference points in this regard. Moreover, it is highlighted that most of these species would also parasitize humans and could be of zoonotic potential transmitting *Rickettsia* spp., *Borrelia* spp., *Bartonella* spp., *Ehrlichia* spp., *Babesia* spp., and several nairo- and flaviviruses. Though this hypothesis still needs investigation. Integration of host association data shows little evidence of host specificity. This study will definitely serve as an important resource for argasids associated with Palearctic bats.

## Soft ticks in China

Another meta-analysis of argasid ticks in China synthesized current knowledge on the species and associated pathogens for this important but neglected region (Chen et al.). Up to 15 argasid species were discussed including from the Argasinae: *Argas assimilis, Argas beijingensis, Argas japonicus, A. persicus, Argas pusillus, A. reflexus, Argas robertsi, Argas sinensis, A. vulgaris, Ornithodoros lahorensis* (also known as *Alveonasus lahorensis*); and from the Ornithodorinae: *Argas vespertilionis* (also known as *Carios vespertilionis*), *Ornithodoros capensis* (also known as *Alectorobius capensis*), *Ornithodoros* (*Ornithodoros*) *huajianensis, Ornithodoros* (*Pavlovskyella*) *papillipes*, and *Ornithodoros* (*Pavlovskyella*) *tartakovskyi*. The study brings to light old Chinese papers (1930s to 1960s) on soft ticks and highlights that the diversity of argasid species in this region could be by far underestimated.

## Salivary gland transcriptomes of soft ticks

Lastly, the study by Reck et al. described the salivary gland transcriptome of *Ornithodoros* (*Pavlovskyella*) *brasiliensis* elucidated by next-generation sequencing. This study contributed a large number of genes from this species including secretory proteins and confirmed that these latter are highly abundant in argasid salivary glands. This gene catalog will be useful to identify toxins involved in toxicosis syndromes caused by this tick species in mammals.

## Conclusions

This special issue highlighted the diversity of subject areas that can be illuminated by the study of argasid species and their biology. It also represents a summary of some areas of interest in contemporary argasid biology and promises that further study into these research lines will expand in the future.

## Author contributions

BM: conceptualization and writing—original draft, and review and editing. JV and SM-L: writing—review and editing. All authors read and approved the final manuscript.

## Conflict of interest

The authors declare that the research was conducted in the absence of any commercial or financial relationships that could be construed as a potential conflict of interest.

## Publisher's note

All claims expressed in this article are solely those of the authors and do not necessarily represent those of their affiliated organizations, or those of the publisher, the editors and the reviewers. Any product that may be evaluated in this article, or claim that may be made by its manufacturer, is not guaranteed or endorsed by the publisher.
